# Pediatric duodenal stenosis caused by posttraumatic mesenteric hematoma managed with a double elementary diet tube

**DOI:** 10.1016/j.tcr.2024.101060

**Published:** 2024-06-05

**Authors:** Hirotaka Shibuya, Keita Sato, Natsuki Hashiba, Yosuke Yamauchi, Yoshihisa Tamura, Shinya Sugimoto, Koji Takahashi

**Affiliations:** a Department of Surgery, Ise Red Cross Hospital, Ise, Mie, Japan; b Department of Gastroenterology, Ise Red Cross Hospital, Ise, Mie, Japan; c Department of Gastroenterology and Hepatology, Mie University Hospital, Tsu, Mie, Japan; d Department of Hapatobiliary Pancreatic and Transplantation Surgery, Mie University Hospital, Tsu, Mie, Japan

**Keywords:** Duodenal trauma, Mesenteric hematoma, Pediatric trauma, Double elementary diet tube, Duodenal stenosis

## Abstract

A 6-year-old male child was admitted to the hospital because of abdominal trauma and acute stomach pain. Computed tomography scan revealed a jejunal mesenteric hematoma and an enhanced intestinal wall compressed by the hematoma. The patient presented with vomiting 10 days after the injury. He underwent upper endoscopy under tracheal intubation and general anesthesia 12 days after the injury. A double elementary diet tube was inserted endoscopically with the tip placed in the jejunum beyond the stenosis and the decompressed portion of the stomach. Stenosis was improving, and the patient was discharged on the 27th day after the injury. In conclusion, a double elementary diet tube can be effective for treating posttraumatic duodenal stenosis in pediatric patients.

## Background

Children have lesser intraabdominal fat than adults. Hence, pediatric abdominal trauma is associated with a higher risk of duodenal injury and a higher incidence of duodenal hematoma than adult abdominal trauma [1]. Posttraumatic duodenal stenosis can be caused by a duodenal intramural hematoma^1–3^. However, this condition [[1], [2], [3]] [[1], [2], [3]] is rare. Herein, we report a case of mesenteric hematoma caused by pediatric abdominal trauma and duodenal stenosis that occurred 10 days after the injury and was treated with a double elementary diet (W-ED) tube.

## Case report

**Fig. 1:**
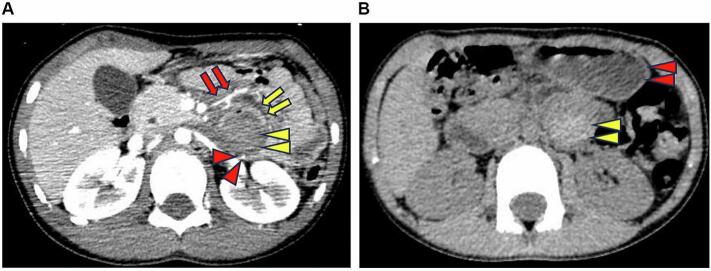
A: Axial contrast-enhanced computed tomography scan performed on the day of injury showed a mesenteric hematoma (yellow arrowhead) compressing the third portion of the duodenum (yellow arrow), the jejunal artery around the hematoma (red arrow), and the inferior mesenteric vein running dorsal to the hematoma (red arrowhead). B: Axial plain computed tomography scan performed on the 10th after the injury showed a mesenteric hematoma of the same size (yellow arrowhead) and gastric dilatation (red arrowhead). (For interpretation of the references to colour in this figure legend, the reader is referred to the web version of this article.)

**Fig. 2:**
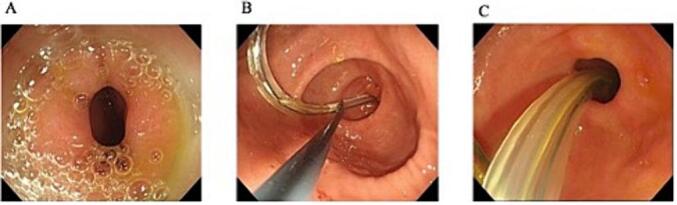
A: Upper endoscopy performed on the 11th day after the injury showed stenosis at the third potion of the duodenum. B: The endoscopic image showed grasping of the double elementary diet tube with grasping forceps and controlling of the feeding tube to pass through the stenosis. C: The endoscopic image showed the double elementary diet tube passing through the stenosis.

**Fig. 3:**
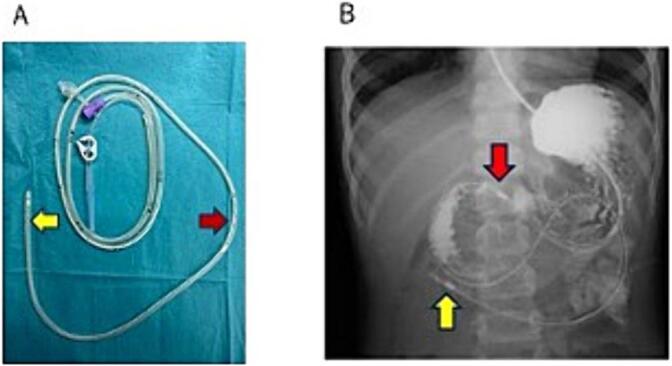
(A)Picture of a double elementary diet tube. The nutrient administration cavity has a hole at the tip(yellow arrow), and the drainage cavity has a drainage hole 40 cm from the tip (red arrow). (B)Abdominal radiographic image after gastrointestinal angiography performed on the 12th day after the injury showed the double elementary diet tube, with the tip placed at the jejunum (yellow arrow) and the drainage marking placed at the stomach (red arrow). (For interpretation of the references to colour in this figure legend, the reader is referred to the web version of this article.)

**Fig. 4:**
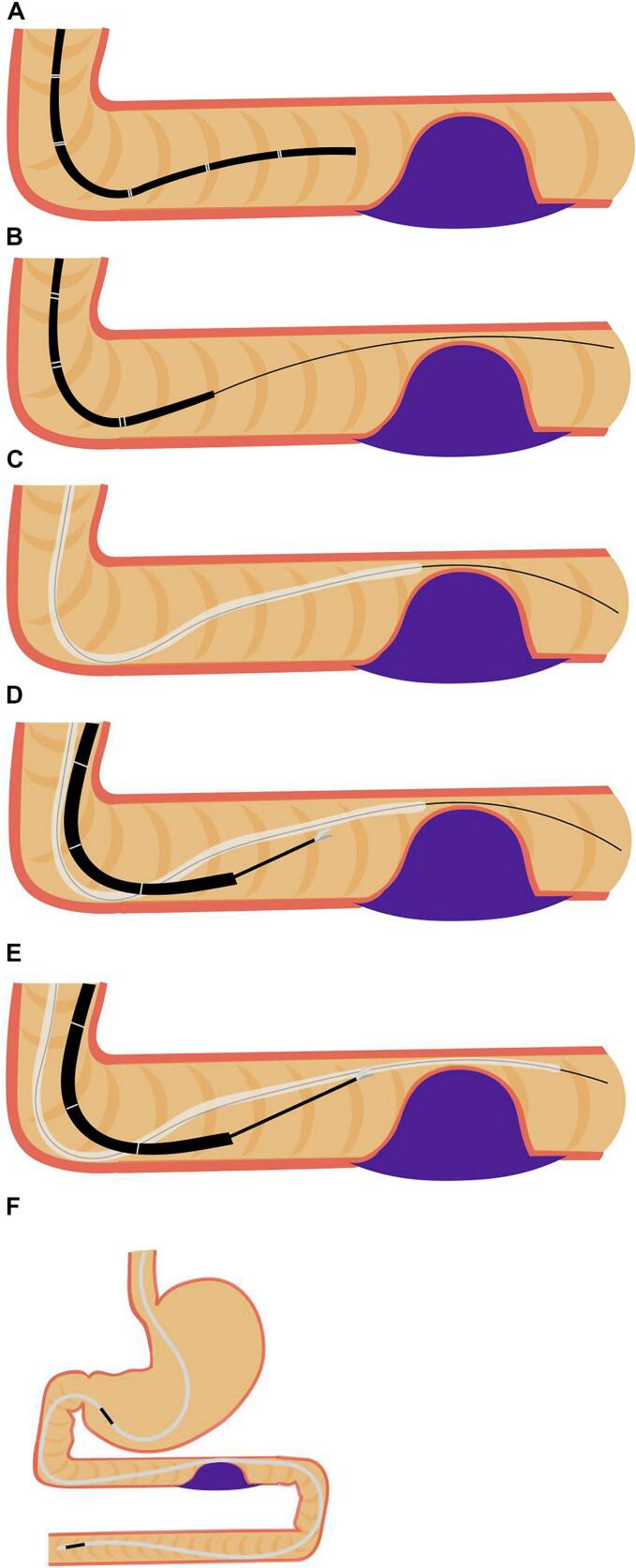
Upper endoscopic images during double elementary diet (W-ED) tube insertion. A: Stenosis of the third portion of the duodenum with transnasal endoscopy. B: Guidewire passing via the stenosis. C: The W-ED tube did not pass through the stenosis with wire guidance. D, E: The W-ED tube was grasped with grasping forceps on transoral endoscopy and guided to the jejunum and passed through the stenosis. F: The feeding hole was placed in the duodenum, and the drainage hole was placed in the stomach.

A 6-year-old male child fell over with a water bottle clutched in his stomach, and he was then admitted to the emergency department with acute abdominal pain. The patient had no history of any disease. His height was 122 cm, and his weight was 21 kg. He presented with tenderness in the whole abdomen. Contrast-enhanced computed tomography (CT) scan of the abdomen revealed a jejunal mesenteric hematoma, and the enhanced intestinal wall was compressed by the hematoma (Fig. 1). He could eat well and was discharged on day 5. At 5 days after he was discharged, he was admitted to the emergency department again due to vomiting and stomach pain. Plain abdominal CT scan revealed a mesenteric hematoma of the same size and gastric dilatation (Fig. 2). He was diagnosed with a jejunal mesenteric hematoma causing stenosis of the third portion of the duodenum. Upper endoscopy was performed under tracheal intubation and general anesthesia on the third day of readmission. A W-ED tube was inserted endoscopically with the tip placed in the jejunum beyond the stenosis and the decompressed portion of the stomach. When passing through the stenosis, the W-ED tube was grasped with endoscopic grasping forceps and guided to the jejunum (Fig. 3, Fig. 4). Intragastric decompression and tube feeding were initiated. Contrast-enhanced imaging studies confirmed improvement in the stenotic area. Oral intake was initiated 7 days after readmission. The W-ED tube was removed, and he was discharged 18 days after readmission.

## Discussion

Posttraumatic duodenal stenosis can be caused by a duodenal intramural hematoma^1–3^. However, this condition [[1], [2], [3]] [[1], [2], [3]] is rare. Previous studies have reported about duodenal stenosis caused by mesenteric hematoma, and it is classified as endogenous,^[^^4^^]^ iatrogenic,^[^^5^^]^ or idiopathic.^[^^6^^]^ There are only two reports about duodenal stenosis caused by traumatic mesenteric hematoma.^[^^7^^,^^8^^]^ Previous reports have shown the development of duodenal stenosis 6 or 8 years after injury.^[^^7^^,^^8^^]^ Nevertheless, in the current case, duodenal stenosis developed 10 days after injury. Hematoma is attributed to the pressure caused by the trauma from the ventral side and the hematoma in the mesentery at the jejunal origin, pinched by the mesentery on the spine by a mechanism similar to that of traumatic duodenal injury.^[^^1^^]^

The common symptoms of jejunal mesenteric hematoma are nonspecific, and they include abdominal pain and nausea. Contrast-enhanced CT scan showed the following characteristic findings: First, the hematoma was located around the jejunal arteries and ventral to the inferior mesenteric vein. Hence, it was not a retroperitoneal hematoma. Second, the contrast-enhanced intestinal wall was compressed, indicating that the hematoma was outside the intestinal tract. The findings revealed a hematoma located in the jejunal mesentery. Upper endoscopy and gastrointestinal angiography are effective in diagnosing duodenal stenosis^4^. On the 2nd day of readmission, upper endoscopy was performed under fluoroscopy to confirm the presence of stenosis in the duodenal third portion. Then, a W-ED tube was placed directly. Patient burden was reduced by performing gastrointestinal angiography, making a stenosis diagnosis, placing a drainage tube, and replacing the feeding tube in a single examination.

Conservative therapy with intragastric drainage and central venous nutrition is the treatment of choice for duodenal stenosis caused by hematoma. Surgical treatment is used if long-term conservative treatment is not successful in improving the condition.^[^^1^^]^ Prolonged nutritional management without intestinal tract and surgical therapy are challenging options for pediatric patients. A W-ED tube has a two-lumen structure. The nutrient administration cavity has a hole at the tip, and the drainage cavity has a drainage hole 40 cm from the tip. The feeding hole is placed in the duodenum or jejunum, and the drainage hole is placed in the stomach to perform the dual functions of feeding and drainage.^[^^6^^]^ Decompression and enteral nutrition can be performed together. Moreover, W-ED is less invasive than surgical treatment. Hence, it can be the primary option for pediatric patients with duodenal stenosis. The use of a W-ED tube in duodenal stenosis caused by hematoma has been reported in one case of idiopathic mesenteric hematoma.^[^^6^^]^ The current case first showed its use in a patient with trauma.

The 16-F W-ED tube is not easy to pass through the stenosis. In this case, the guidewire passed through the stenosis. However, the W-ED tube did not. We could pass through the stenosis by grasping the W-ED tube with biopsy forceps and controlling the tube to feed it. This method can be effective when passing the W-ED tube through the stenosis if the tube does not pass via guide wire guidance.

## Conclusion

Herein, we present a rare case of traumatic jejunal mesenteric hematoma and duodenal stenosis. A W ED tube can be an effective tool, and its placement can be the primary option for the treatment of pediatric traumatic duodenal stenosis.

## Presentation

The content of this paper was presented at The 51st Annual Meeting of the Japanese Association for Acute Medicine in 2022.

## CRediT authorship contribution statement

**Hirotaka Shibuya:** Writing – original draft. **Keita Sato:** Writing – review & editing. **Natsuki Hashiba:** Investigation. **Yosuke Yamauchi:** Investigation. **Yoshihisa Tamura:** Investigation. **Shinya Sugimoto:** Writing – review & editing. **Koji Takahashi:** Supervision.

## Declaration of competing interest

The authors declare that they have no known competing financial interests or personal relationships that could have appeared to influence the work reported in this paper.

## Data Availability

All data generated or analyzed during this study are included in this published article.
